# A Population-Level Assessment of Smoking Cessation following a Diagnosis of Tobacco- or Nontobacco-Related Cancer among United States Adults

**DOI:** 10.1155/2021/6683014

**Published:** 2021-01-19

**Authors:** Richard S. Matulewicz, Marc A. Bjurlin, Zachary Feuer, Danil V. Makarov, Scott E. Sherman, Joy Scheidell, Maria R. Khan, Omar El-Shahawy

**Affiliations:** ^1^New York University School of Medicine, Department of Population Health, USA; ^2^New York University School of Medicine, Department of Urology, USA; ^3^VA New York Harbor Healthcare System, USA; ^4^University of North Carolina, Department of Urology, Lineberger Comprehensive Cancer Center, USA; ^5^New York University School of Global Public Health, Division of Global Health, USA

## Abstract

**Introduction:**

Smoking cessation after a cancer diagnosis can significantly improve treatment outcomes and reduce the risk of cancer recurrence and all-cause mortality.

**Aim:**

We sought to measure the association between cancer diagnosis and subsequent smoking cessation.

**Methods:**

Data was sourced from the Population Assessment of Health and Tobacco (PATH) study, a representative population-based sample of United States adults. Our analytic sample included all adult smokers at Wave I, our baseline. The exposure of interest was either a tobacco-related cancer diagnosis, nontobacco-related cancer diagnosis, or no cancer diagnosis (the referent) reported at Wave II or III. The primary outcome was smoking cessation after diagnosis, at Wave IV. *Results/Findings*. Our sample was composed of 7,286 adult smokers at the baseline representing an estimated 40.9 million persons. Smoking cessation rates after a diagnosis differed after a tobacco-related cancer (25.9%), a nontobacco-related cancer (8.9%), and no cancer diagnosis (17.9%). After adjustment, diagnosis with a tobacco-related cancer was associated with a higher odds of smoking cessation (OR 1.83, 95% CI 1.00-3.33) compared to no cancer diagnosis. Diagnosis with a nontobacco-related cancer was not significantly linked to smoking cessation (OR 0.52, 95% CI 0.48-1.45).

**Conclusion:**

Diagnosis with a tobacco-related cancer is associated with greater odds of subsequent smoking cessation compared to no cancer diagnosis, suggesting that significant behavioral change may occur in this setting.

## 1. Introduction

Smoking causes over 500,000 deaths each year in the United States, 40% of which are cancer-related [1]. Overall, smoking cessation reduces the risk of cancer recurrence and the incidence of second malignancies; it is estimated to lower all-cause mortality rates by 30-40% [2, 3]. However, 50-75% of adults may continue to smoke after their cancer diagnosis despite the increased risk of cardiopulmonary events, attenuated responses to systemic treatment, and exacerbated treatment side effects [4–8]. Conceptually, a cancer diagnosis is thought to be a “teachable moment” and an opportunity for substantive lifestyle changes, which may influence smoking quit behavior [9–13]. However, prior studies have reported significant variation in rates of smoking cessation after a cancer diagnosis [5, 7, 14]. Nevertheless, most of these studies have mostly evaluated the association of cancer diagnosis collectively on quit behavior without adjusting for the cancer type, whether it is tobacco-related or nontobacco-related cancer.

The existing evidence on the impact of cancer diagnosis on quit behavior has several challenges. The studies are either retrospective, cross-sectional, or limited to cohorts of long-term survivors, which can complicate accurate event recall and makes assessment of temporal association challenging and does not eliminate reverse causality [5, 7, 14]. Understanding the impact of new cancer diagnosis and other related factors on a population level is essential to inform the delivery of evidence-based smoking cessation interventions during routine oncology practice.

The authors could not find any contemporary longitudinal population-based studies of the prevalence and correlates of smoking cessation after a new cancer diagnosis among United States adults. To address this gap in the literature, the objective of this study is to evaluate the association of a new cancer diagnosis with subsequent smoking cessation using longitudinal population-level data in the US. We hypothesize that adults diagnosed with cancer will be more likely to quit smoking than those not diagnosed with cancer and that cancer type (tobacco- or nontobacco-related) may influence this association. We also sought to better understand the correlates of smoking cessation after a cancer diagnosis and explore the use of quit aids such as supportive therapies, nicotine replacement, and the use of alternative tobacco products to better understand behaviors related to smoking cessation.

## 2. Methods

### 2.1. Study Design/Data Source

We used prospectively collected survey data from the PATH (Population Assessment of Tobacco and Health) study. The PATH study is an ongoing nationally representative prospective longitudinal cohort study sponsored by the NIH and FDA which began in 2013. Baseline and follow-up information on tobacco use patterns (initiation and cessation behaviors) among participants as well as tobacco- and nontobacco-related health outcomes such as cardiovascular events and cancer diagnoses is collected [15]. Complete information regarding participant sampling, drop out and replenishment strategies, data collection, and collection period timing can be found at the study website and in the public use file [15]. Longitudinal weighting was used to account for participant dropout and replenishment thus allowing weights to reflect a representative population. The reporting of this study is in accordance with the Strengthening the Reporting of Observational Studies in Epidemiology (STOBE) guidelines [16]. This study uses publicly available, deidentified data and does not require IRB approval.

The present analysis capitalizes on the longitudinal cohort study design of PATH to assess the proportion of adult smokers diagnosed with cancer who quit smoking after diagnosis and to determine factors related to their odds of quitting. Survey data included in this study was collected in the first four “waves” which began September 2013 (Wave I) and was completed in January 2018 (Wave IV). Each survey “wave” represents approximately 1 year of elapsed time from the prior wave. All data was self-reported other than biometric information such as BMI, which was measured. The exact survey instrument question language corresponding to pertinent variables can be found in Supplementary 1: Appendix Table. The analytic sample for this study is adults that responded to surveys for all four waves and reported being current, established smokers at baseline (Wave I). Participants were considered a current established smoker at baseline if they reported smoking cigarettes either some days or every day at the time of the Wave I survey and they had smoked >100 cigarettes in their lifetime.

### 2.2. Exposure

**Figure 1:**
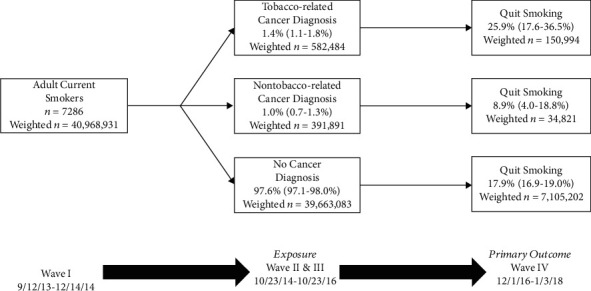
Study schema demonstrating the survey waves/dates that correspond with our exposure of interest (tobacco- or nontobacco-related cancer diagnosis versus no cancer diagnosis (the referent) at Wave II/III) and our primary outcome (smoking cessation at Wave IV relative to Wave I) for an analytic sample of adult current smokers at baseline (Wave I). Percentages are population prevalence estimates with 95% confidence intervals. Data collection for each wave was performed over the course of the listed time period, but elapsed time between waves for each participant was roughly 1 year from the prior wave.

Our initial exposure of interest was a cancer diagnosis reported at Wave II or Wave III. The survey instrument question at Waves II and III asked, “In the past 12 months, have you been told by a doctor, nurse, or other health professionals that you had cancer?” Therefore, the reported diagnosis was in the 24-month period between after the Wave I survey and before the Wave III survey (Figure 1). Based on reported diagnoses at Waves II and III, we categorized cancer type (tobacco-related vs. nontobacco-related) as a three-level exposure variable (tobacco-related cancer diagnosis, nontobacco-related cancer diagnosis, or no cancer diagnosis). According to the PATH study investigator designation (Health et al., 2019), a reported cancer type was categorized to (a) tobacco-related: bladder, cervix, colon, esophagus, kidney, larynx, liver, lung, mouth, pancreas, rectum, stomach, and throat or (b) nontobacco-related: blood, bone, brain, breast, gallbladder, leukemia, lymphoma, melanoma, nervous system, ovarian, prostate, nonmelanoma, unknown skin, soft tissue, testicular, thyroid, and uterine.

### 2.3. Main Outcome

Our main outcome was smoking cessation at Wave IV relative to baseline (Wave I) smoking status. This outcome was based on a “no” response to the survey question: “In the past 12 months, have you smoked a cigarette, even one or two puffs?” Participants who quit at Waves II or III and relapsed at Wave IV were considered smokers (and not having quit) as of Wave IV.

### 2.4. Covariates

Age was dichotomized as either 18-54 years or 55 and older since only age was included as a categorical variable in the public use dataset, thus precluding our ability to assess as a continuous variable. Additional covariates included body mass index (BMI; under/normal weight defined as BMI < 25 versus overweight/obese, BMI ≥ 25), marital status (never married, previously married defined as widowed, separated, divorced versus married), US census region derived from participant home zip code (Northeast, Midwest, South, and West), poverty level (at or below poverty level versus above), education level (<high school, finished HS/completed some college, and finished college/more advanced degree), and comorbidity burden based on a modified version of the Charlson-Deyo score [17] which includes hypertension, hyperlipidemia, congestive heart failure (CHF), prior stroke, heart disease, prior heart attack, prior history of cancer, COPD, diabetes, and peptic ulcer disease in which the presence of each comorbidity was considered a single “point” towards the score (having 1+ comorbidity versus none).

Tobacco dependence was determined by time to first cigarette (TTFC) from waking, which is an established proxy measure that has a high predictive validity compared to using the total Fagerström Test for Nicotine Dependence score [18]. Tobacco dependence was categorized to within 5 minutes, 6-30 minutes, 31-60 minutes, and 60+ minutes. Participants were considered to be using other tobacco products (OTPs) at baseline (Wave I) if they reported current and established (meeting minimal duration and quantity minimums, see Supplementary 1: Appendix Table) use of e-nicotine products (e-cigarettes, e-cigars, and e-hookah), cigarillos, cigars, pipes, hookah, snus, or smokeless/chewable tobacco. A composite variable for any OTP use was created and dichotomized (yes, no). Prior attempts to quit smoking at the time of Wave I were used as a proxy measure for baseline self-efficacy and was defined as a binary variable with “yes” indicating any attempt to quit entirely or an attempt at cutting back with the goal of ultimately quitting within the past 12 months.

Among adults who quit smoking or attempted to quit in the prior year (at Wave IV), we explored use of “quit aids” such as behavioral counseling, support of family and friends, nicotine replacement products, and prescription medications (such as bupropion or varenicline) with descriptive statistics. Additionally, use of other tobacco products as a substitute for cigarette smoking was assessed within this group.

### 2.5. Data Analysis

Data analysis was conducted from September 2019 through August 2020. The survey replicate weight, variance estimation method, BRR-Fay replicate weights, and Fay's factor were used per PATH study recommendations and reflective of a longitudinal study that induces respondents to all four consecutive waves [15]. We used survey commands in Stata Version 16.0 (StataCorp, College Station, Texas) to account for stratification, clustering, and unequal selection probabilities, yielding nationally representative estimates.

We calculated weighted percentages of sociodemographic, disease severity, and tobacco use variables. We calculated unadjusted and adjusted odds ratios (ORs) and 95% confidence intervals (CIs) for associations between cancer diagnosis status (tobacco-related cancer diagnosis, nontobacco-related cancer diagnosis versus no cancer diagnosis, the referent) at Wave II/III with smoking cessation at Wave IV, adjusted for several demographic, comorbidity, and tobacco dependence variables that were selected *a priori* based on prior literature. Prior to inclusion in the multivariable model, variables were tested for interaction, linearity in the log odds, and collinearity using Pearson's correlation matrix (Supplementary 2). Sensitivity analyses were performed using alternative outcomes that included both smoking cessation at Wave IV and attempts to quit completely at Wave IV to further test the exposure/outcome association. We also tested whether including a prior history of cancer (at “baseline,” Wave 1) as a separate variable had a significant interaction or association with our primary outcome as an additional sensitivity analysis. All statistics were performed with a two-sided significance set to be <0.05.

## 3. Results

### 3.1. Demographics and Baseline Variables

In total, 21,285 adult smokers and nonsmokers participated in all four waves of the survey and were weighted to represent a population-level estimate of American adults. By design, the survey weights accounted for loss to follow-up at each wave and were adjusted to assure representative sampling weights for the US population at Wave IV. Participants were excluded for being a nonsmoker at Wave I (raw *n* = 13,946) and for having incomplete smoking status at Wave I or IV (Wave I raw *n* = 49, Wave IV = 17). Therefore, 7,286 adults met the criteria as current established smokers at baseline (Wave I) and comprised our analytic sample, which translated to an estimated 40.9M (95% CI 39.7M-42.2M) US adults.

Among the adult smokers in our sample, 70.0% were white, 78.4% were younger than 55, and 54.7% were male. Most reported being healthy (65.0% free of any comorbidities), but 63.4% were overweight or obese. An estimated 4.9% of patients had a prior history of cancer at baseline (Supplementary 2). Regarding tobacco use and dependence, 47.6% of participants reported making at least one attempt to quit cigarettes entirely in the 12 months preceding the Wave I survey, and 23.7% reported tobacco polyuse, consuming at least one additional tobacco or e-nicotine product other than cigarettes at baseline. Respondents reported using their first cigarette less than 5 minutes (23.1%) or within 6-30 minutes (37.0%) of waking each morning. No baseline covariates demonstrated significant collinearity. Full baseline sociodemographic data can be found in Table 1.

### 3.2. Relationship between a New Cancer Diagnosis and Smoking Cessation

During our exposure period (Waves II and III), 1.4% (weighted *n* = 582,484) and 1.0% (weighted *n* = 391,891) reported being newly diagnosed with a tobacco- or nontobacco-related cancer, respectively. At Wave IV, 17.8% (weighted *n* = 7.4M) of all smokers at baseline (Wave I) had quit (Figure 1). The proportion of smokers who quit varied among those diagnosed with a tobacco-related (25.9%, weighted *n* = 150,994) and nontobacco-related (8.9%, weighted *n* = 34,821) cancer compared to those who were not diagnosed with cancer (17.9%, weighted *n* = 7,105,202). Unadjusted bivariate analysis demonstrated a nonsignificant association between quitting and being diagnosed with a tobacco-related cancer (unadjusted OR 1.60, 95% CI 0.97-2.66) and being diagnosed with a nontobacco-related cancer (unadjusted OR 0.45, 95% CI 0.17-1.17) relative to smokers that did not report a cancer diagnosis (Table 2). However, after adjusting for all demographic, comorbidity, and tobacco dependence covariates, there was an association between reporting a tobacco-related cancer diagnosis and an increased odds of subsequent smoking cessation (OR 1.83, 95% CI 1.00-3.33) but not with a nontobacco-related cancer diagnosis (OR 0.52, 95% CI 0.19-1.44) (Table 2). However, when assessing the association of a new diagnosis with either quitting or attempting to quit, no association was present for either a tobacco-related cancer diagnosis (OR 1.05, 95% CI 0.62-1.78) or a nontobacco-related diagnosis (OR 0.83, 95% CI 0.48-1.45). A prior history of cancer at baseline did not have a significant association with our outcome when included as a unique variable (OR 0.99, 95% CI 0.70-1.40).

### 3.3. Exploratory Outcomes: Use of “Quit Aids”

Among smokers that reported successful smoking cessation or attempted to quit in the year preceding the Wave IV survey, the most commonly reported “quit aid” was the support of family and friends, used by 32.1%, followed by 9.1% who used nicotine replacement products such as gum or lozenges and 3.9% who used prescription medications (Table 3). Use of other tobacco products as a means of quitting or attempting to quit smoking was reported in 16.1% of people; among those, e-nicotine product use was reported by 81.7%.

## 4. Discussion

Our results demonstrate that more US adults quit smoking after a tobacco-related cancer diagnosis compared to those not diagnosed with cancer. Although smoking cessation treatment after a cancer diagnosis is a recommended essential component of comprehensive cancer care, there is a large potential to improve since only approximately 25% of patients with a tobacco-related cancer quits after diagnosis. Patient awareness of the connection between smoking and cancer for tobacco-related diagnoses may facilitate behavioral change. Similarly, there seems to be a far greater missed opportunity to help patients with nontobacco-related cancers quit smoking. Improving the approach to and delivery of effective smoking cessation treatment, potentially targeting informing patients of the benefits of quitting smoking irrespective of the cancer type, may improve the overall smoking cessation rates among cancer survivors.

Prior studies assessing smoking status among patients diagnosed with cancer could not address subsequent effects on smoking cessation because they were either cross-sectional, retrospective series, or among long-term survivors. A population-based study using the NHANES database (1999-2008) demonstrated that 73.5% (932/1267) of cancer survivors who had ever smoked reported having quit smoking at various points after their diagnosis [19]. Two additional population-based studies using data from the NCI's HINTS survey (2003-2007) and the CPS-II Nutrition Cohort (1992-2009) demonstrated discordant findings [7, 20]. Variation in findings among studies may relate to general temporal trends in smoking cessation, recall bias, and differences in study design relating to the timing and sequence of exposure and outcome. A pooled analysis of 1301 patients from a systematic review of ten RCTs and three prospective cohort studies reported 17.6% (229/1301) of patients quitting between six weeks and six months, most of which were verified biochemically [21]. These numbers are likely more concordant with our findings due to similarities in event-outcome timing and study design and may better reflect a true estimate of the short term (~1 year) quit rates among cancer survivors. However, a major strength in our study is that it represents the longitudinal population-level estimates for smoking cessation among cancer survivors in the US.

It is postulated that a cancer diagnosis is a “teachable moment” and prime for a smoking cessation intervention [7, 9, 10]. Thus, NCI now recommends tobacco cessation counseling for all cancer survivors [22]. It is particularly important for medical specialties that treat tobacco-related cancers to understand and implement these interventions into their clinical practice and improve the process by which they accomplish this as a prime opportunity for implementation science research. Our study also underscores the importance of informing cancer survivors of the benefits of smoking cessation, regardless of their cancer type. There has been little differentiation between the impact of tobacco- and nontobacco-related cancer diagnosis on smoking cessation. Our findings suggest that there may be a misconception among cancer survivors who are diagnosed with a nontobacco-related cancer that smoking cessation may not be of benefit. However, future studies should test this assumption to help design better communication strategies targeting cancer survivors who smoke and eliminate any misinformation they may have.

Utilization of evidence-based cessation aids was low among those who managed to quit or attempted to quit. When considering the cohort who were able to quit, most relied on family and friends for support, which could be part of the stronger prohealth behavior support network that forms around a cancer survivor in such a demanding health situation. While such support is very important for quit success, there were far fewer smokers who relied on nicotine replacement therapies (NRT) (e.g., lozenges, gum, or patches) or pharmaceutical agents. This reflects a potential lack of effective counseling or involvement from medical providers such as oncologists and other cancer specialists due to their lack of comfort or familiarity with prescribing these agents and is a potential opportunity for improvement [23, 24]. Cancer survivors benefit greatly from smoking cessation; as such, intensive treatment protocols that consistently utilize cessation medications may enhance quit success in a period where smoking cessation becomes of paramount importance. These findings may inform how we tailor smoking cessation interventions for cancer survivors by engaging social or behavioral support networks that form around the cancer survivor in addition to other traditional tobacco cessation strategies. Additionally, in lieu of quitting combustible cigarettes, many adults alternatively used e-nicotine products. Given the uncertainty surrounding the safety of e-products, it is unclear whether this may be an effective risk reduction strategy or exposure to persistently harmful products. This is especially important since most smokers who use these products continue to do so instead of quitting nicotine products altogether [25].

Prior to this study, we could not find any contemporary US population-based longitudinal assessments of the prevalence and correlates of smoking cessation after a new cancer diagnosis. Additionally, a major strength of our study is evaluating cancer diagnosis by cancer type. However, there are limitations to our study. We attempted to account rigorously for the temporality of both the cancer diagnosis exposure and the smoking cessation outcome by firmly establishing the sequence with exposure (diagnosis at Wave II or III) and outcome at Wave IV. However, it is possible that patients may have quit around the time of diagnosis (i.e., shortly before, at Wave II or III) when adverse health effects started to become apparent from a cancer prodrome rather than immediately afterwards. Nevertheless, the exact sequence of events is less clinically relevant since there are well-established benefits to smoking cessation at any point before or after diagnosis. Importantly, survey responses are subject to recall bias. Our study lacked biochemical confirmation of smoking abstinence and therefore may overreport cessation. However, we attempted to control for this by requiring two separate and concordant survey responses and only considering strict reported abstinence as evidence of quitting, yielding a relatively robust association [21]. We also do not have data on long-term sustained cigarette smoking abstinence beyond 1 year. Cancer type was self-reported so misclassification or inaccurate reporting was possible. The cancer type was also designated as tobacco- versus nontobacco-related based on PATH study criteria without added granularity of specific diagnosis types. For example, while leukemias are largely nontobacco-related, acute myeloid leukemia is listed as tobacco-related in the Surgeon General's report. Finally, there is the possibility of either residual confounding or reverse causality from patients with cancer not being able to smoke due to complications of their cancer. Statistically, despite being a population-based study of over 30,000 participants, we were limited by a small number of exposures (cancer diagnoses) and few patients who quit smoking (outcome). This may have been partially responsible for our adjusted analysis meeting standards of statistical significance while unadjusted estimates of the odds of quitting among those adults with tobacco-related cancers did not. The representative sampling study design and our sample's population estimate well reflect the estimated 34.2 million smokers in America as of 2014 [1]. Critical findings include low overall rates of smoking cessation among those diagnosed with cancer, which may represent a missed opportunity for delivering effective smoking cessation treatment.

## 5. Conclusion

In this population-based study of US smokers, adults diagnosed with tobacco-related cancers were more likely to quit smoking than those not diagnosed with cancer. There is considerable potential to increase the proportion of patients who quit smoking after a cancer diagnosis, and our findings may highlight a potential opportunity to leverage the time around a cancer diagnosis for the delivery of smoking cessation treatment to patients diagnosed with cancer. There is particularly a missed opportunity in engaging cancer survivors of nontobacco-related cancer in smoking cessation treatment. Further investigation is needed to assess why adults with nontobacco-related cancers continue to smoke cigarettes after diagnosis.

## Figures and Tables

**Table 1:** Sociodemographic and tobacco use characteristics at baseline: Population Assessment of Tobacco or Health survey.

	Categories	Raw *N* (7,286)	Weighted *N* (40,968,509)	Weighted % (95% confidence interval)
Age	18-54	5,874	32,096,414	78.4 (77.1-79.5)
	54+	1,410	8,862,095	21.6 (20.5-22.9)
Sex	Female	3,716	18,545,873	45.3 (44.1-46.5)
	Male	3,570	22,423,058	54.7 (53.5-56.0)
Modified Charlson-Deyo score	0 (healthy)	4,796	26,550,304	65.0 (63.6-66.3)
	1+ (comorbid)	2,469	14,310,104	35.0 (33.7-36.4)
Body mass index (BMI)	Under/normal weight	2,646	14,986,556	36.6 (35.2-38.0)
	Overweight/obese	4,640	25,982,375	63.4 (62.0-64.8)
Marital status	Never married	2,739	14,567,372	35.7 (34.2-37.2)
	Married	2,541	14,745,489	36.1 (34.7-37.6)
	Divorced/separated/widowed	1,980	11,512,986	28.2 (27.0-29.5)
Race	White non-Hispanic	4,710	28,239,696	70.0 (68.8-71.2)
	Black non-Hispanic	1,037	5,272,514	13.1 (12.3-13.9)
	Hispanic	945	4,517,035	11.2 (10.5-11.9)
	Other	488	2,310,745	5.7 (5.2-6.3)
Census region	Northeast	1,062	7,147,997	17.5 (15.6-19.0)
	Midwest	2,074	10,049,180	24.5 (22.9-26.2)
	South	2,814	16,273,532	39.7 (37.8-41.7)
	West	1,336	7,498,221	18.3 (16.7-20.0)
Poverty status	Below poverty level	3,131	15,986,300	40.0 (38.3-41.7)
	At or above poverty level	4,004	23,976,966	60.0 (58.3-61.7)
Highest level of education	Did not finish high school	1,251	6,475,524	15.9 (15.1-16.7)
	High school or some college	5,236	29,638,422	72.7 (71.7-73.7)
	College or more	773	4,666,144	11.4 (10.7-12.2)
Prior attempts to quit	None	3,735	21,485,239	52.4 (51.0-53.8)
	One or more	3,551	19,483,692	47.6 (46.2-49.0)
Other tobacco use	No	5,262	29,878,025	76.3 (75.0-77.5)
	Yes	1,715	9,281,606	23.7 (22.5-25.0)
Time to first cigarette from waking (minutes)	Less than 5	1,649	9,324,791	23.1 (21.8-24.4)
	6-30	2,683	14,966,354	37.0 (35.8-38.2)
	31-60	1,313	7,441,155	18.4 (17.4-19.4)
	60 or more	1,557	8,699,453	21.5 (20.3-22.8)
Cancer diagnosis (Waves II or III)	Nontobacco-related cancer	69	391,891	1.0 (0.7-1.3)
	Tobacco-related cancer	103	582,484	1.4 (1.1-1.8)
	No cancer diagnosis	7,058	39,663,083	97.6 (97.1-98.0)

Note: data missing for age category (*n* = 2), modified Charlson-Deyo score (*n* = 21), marital status (*n* = 26), race (*n* = 106), poverty level (*n* = 151), education (*n* = 26), other tobacco product use (*n* = 309), time to first cigarette (*n* = 84), and cancer diagnosis (*n* = 56).

**Table 2:** Survey weighted estimates of the total population, proportion, and unadjusted and adjusted odds of smokers (at Wave I) who quit smoking (at Wave IV) for each covariate and for our 3-level cancer diagnosis exposure at Wave II/III.

	Categories	Weighted % who quit	Weighted *N* who quit	Unadjusted odds of quitting (95% confidence interval)	Adjusted odds of quitting (95% confidence interval)
Cancer diagnosis (at Wave II/III)	No cancer diagnosis	17.9	7,105,202	REF	REF
	Tobacco-related	25.9	150,994	1.60 (0.97-2.66)	1.83 (1.00-3.33)
	Nontobacco-related	8.9	34,821	0.45 (0.17-1.17)	0.52 (0.19-1.44)
Age	18-54	17.9	5,756,737	REF	
	54+	17.5	1,547,420	0.97 (0.80-1.17)	1.19 (0.95-1.49)
Sex	Female	16.6	3,081,732	REF	REF
	Male	18.9	4,226,821	1.17 (1.01-1.34)	1.07 (0.91-1.25)
Modified Charlson-Deyo score	0 (healthy)	18.6	4,933,096	REF	REF
	1+ (comorbid)	16.6	2,369,120	0.87 (0.75-1.01)	0.92 (0.78-1.08)
Body mass index	Under/normal weight	17.2	2,583,418	REF	REF
	Overweight/obese	18.2	4,725,135	1.07 (0.93-1.23)	1.03 (0.88-1.22)
Marital status	Never married	18.2	2,650,457	REF	REF
	Married	20.5	3,017,606	1.16 (0.98-1.36)	1.11 (0.92-1.34)
	Divorced/separated/widowed	17.9	7,288,364	0.74 (0.62-0.87)	0.84 (0.71-1.00)
Race	White non-Hispanic	17.8	5,021,251	REF	REF
	Black non-Hispanic	14.4	761,798	0.78 (0.61-1.00)	0.87 (0.67-1.12)
	Hispanic	21.6	974,255	1.27 (1.05-1.54)	1.00 (0.79-1.27)
	Other	21.1	486,564	1.23 (0.87-1.75)	0.99 (0.69-1.43)
Census region	Northeast	16.1	1,149,232	REF	REF
	Midwest	18.6	1,865,288	1.19 (0.96-1.47)	1.17 (0.93-1.47)
	South	16.8	2,738,841	1.06 (0.84-1.33)	1.01 (0.79-1.29)
	West	17.8	7,308,553	1.37 (1.08-1.73)	1.65 (0.89-1.52)
Poverty status	Below poverty level	14.4	2,294,552	REF	REF
	At or above poverty level	20.4	4,891,807	1.53 (1.33-1.76)	1.26 (1.07-1.49)
Highest level of education	Did not finish high school	13.0	840,307	REF	REF
	High school or some college	17.1	5,054,413	1.38 (1.10-1.73)	1.30 (1.01-1.67)
	College or more	29.3	1,364,992	2.77 (2.07-3.71)	1.94 (1.44-2.62)
Prior attempts to quit	None	15.9	3,415,793	REF	REF
	One or more	20.0	3,892,760	1.32 (1.14-1.53)	1.35 (1.15-1.58)
Other tobacco product use	No	16.6	4,947,830	REF	REF
	Yes	22.2	2,064,736	1.44 (1.24-1.68)	1.55 (1.31-1.84)
Time to first cigarette from waking (minutes)	Less than 5	8.9	827,402	REF	REF
	6-30	14.7	2,202,408	1.77 (1.46-2.15)	1.75 (1.40-2.19)
	31-60	18.2	1,356,165	2.29 (1.86-2.81)	2.13 (1.66-2.72)
	60 or more	30.8	2,677,836	4.57 (3.69-5.65)	4.23 (3.32-5.40)

**Table 3:** Description of quit aids used among quitters or those who attempted quitting in the past 12 months prior to Wave IV survey (*n* = 2,345, weighted *n* = 12,776,935).

Quit aid used^a^	Survey weighted % (95% confidence interval)
Support of family and friends	32.1 (30.1-34.2)
Formal counseling (behavioral or telephone, web-based) or self-help materials	9.0 (7.7-10.5)
Nicotine replacement product	9.1 (8.3-10.0)
Prescription medication	3.9 (3.4-4.5)
Other tobacco product (any)	16.1 (14.5-17.9)

^a^Quit aid categories are not mutually exclusive.

## Data Availability

Data are publicly available via the Population Assessment of Tobacco and Health (PATH) study. PATH is a national longitudinal study of tobacco use and how it affects the health of people in the United States. People from all over the country take part in this study. The PATH study which started in 2013 is the first large research effort undertaken by the National Institutes of Health (NIH) and the Food and Drug Administration (FDA) since Congress gave FDA authority to regulate tobacco products in 2009, website: https://pathstudyinfo.nih.gov/UI/HomeMobile.aspx.

## Supplementary Materials

Supplementary 1 Appendix Table. Variable details and corresponding survey instrument language including possible responses.

Supplementary 2 Supplemental Table 1: correlation coefficient matrix for included covariates. Supplemental Table 2: disaggregated comorbid conditions at baseline (Wave I). Supplemental Table 3: disaggregated other tobacco product (OTP) use at baseline (Wave I).
