# Supplementary material for: A Population-Level Assessment of Smoking Cessation following a Diagnosis of Tobacco- or Nontobacco-Related Cancer among United States Adults
Source: J Smok Cessat. 2021 Jan 19;2021:6683014. doi: 10.1155/2021/6683014 (PMC8279190; doi:10.1155/2021/6683014)
Supplement: Supplementary 1 — Appendix Table. Variable details and corresponding survey instrument language including possible responses. [file 6683014.f1.docx]

|  | **Wave** | **Question** | **Details** | **Responses** | **Asked of:** |
| --- | --- | --- | --- | --- | --- |
| **Smoking Status*** | All | R0XR_A_CUR_ESTD_CIGS | Do you now smoke cigarettes | Every day, some days, not at all | All |
|  |  |  |  |  |  |
| **Cancer Diagnosis** | 1 | R0X_AX0144_NB | Have you ever been told by a doctor, nurse or other health professional that you had cancer? | Yes, no | All patients at “baseline” who enter the study |
|  | 2-4 | R0X_AX0144_NB | In the past 12 months, have you been told by a doctor, nurse or other health professional that you had cancer | Yes, no | All patients each year / wave |
|  | All | R0X_A_AX0145 | “What kind of cancer?” | bladder, cervix, colon, esophagus, kidney, larynx, liver, lung, mouth,  pancreas, rectum, stomach, and throat; blood, bone, brain, breast, gallbladder, leukemia, lymphoma,  melanoma, nervous system, ovarian,  prostate, non-melanoma, unknown skin, soft tissue, testicular, thyroid,  uterine, or other | If responded “yes” to told you had cancer (as above) |
|  |  |  |  |  |  |
| **Quit help (Table 4)** | 4 | R04_AN0225_X | Thinking back to when you quit, did you use a different tobacco product to help you quit? | No, Yes to each individual X (where X=unique OTP) | Adult respondents who are past 12 month non-electronic tobacco quitters or quit attempters and tried to completely quit at least one time in the past 12 months or did not know or refused to report how many times they tried to completely quit in the past 12 months. |
|  | 4 | R04_AN0215 | Thinking back to when you quit, did you use any of the following to help you: counseling, a telephone help line or quit line, books, pamphlets, videos, a quit tobacco clinic, class, or support group, or an internet or web-based program? | Yes, no | Adult respondents who are past 12 month non-electronic tobacco quitters or quit attempters and tried to completely quit at least one time in the past 12 months or did not know or refused to report how many times they tried to completely quit in the past 12 months. |
|  | 4 | R04_AN0210 | Thinking back to when you quit, did you rely on the support of friends and family to help you? | Yes, no | Adult respondents who are past 12 month non-electronic tobacco quitters or quit attempters and tried to completely quit at least one time in the past 12 months or did not know or refused to report how many times they tried to completely quit in the past 12 months. |
|  | 4 | R04_AN0155 | Did you use the nicotine patch, gum, inhaler, nasal spray, lozenge or pill to quit completely? | Yes, no | Adult respondents who are past 12 month non-electronic tobacco quitters or quit attempters and used nicotine replacement therapy in the past 12 months and used nicotine replacement therapy to help them quit. |
|  | 4 | R04_AN0180 | Thinking back to when you quit in the past 12 months, did you use Chantix, varenicline, Wellbutrin, Zyban, or bupropion? | Yes, no | Adult respondents who are past 12 month non-electronic tobacco quitters or quit attempters and used a prescription drug in the past 12 months. |
|  |  |  |  |  |  |
| **Other Tobacco Products determination*** | 1 | R01R_A_CUR_ESTD_ECIG, R03R_A_CUR_ESTD_EPRODS | Adult respondents who have ever used any electronic nicotine products, have ever used them fairly regularly, and currently use them every day or some days. | Yes, no | All who have ever used each respective product |
|  |  | R0XR_A_CUR_ESTD_GTRAD | Adult respondents who have ever smoked a cigar, have smoked at least one cigar product fairly regularly, and currently smoke every day or some days | Yes, no | All who have ever used each respective product |
|  |  | R0XR_A_CUR_ESTD_GRILLO | Adult respondents who have ever smoked a cigarillo, have ever smoked them fairly regularly, and currently smoke every day or some days. | Yes, no | All who have ever used each respective product |
|  |  | R0XR_A_CUR_ESTD_GFILTR | Adult respondents who have ever smoked a filtered cigar, have ever smoked them fairly regularly, and currently smoke every day or some days. | Yes, no | All who have ever used each respective product |
|  |  | R0XR_A_CUR_ESTD_PIPE | Adult respondents who have ever smoked a pipe, have ever smoked fairly regularly, and currently smoke every day or some days. | Yes, no | All who have ever used each respective product |
|  |  | R0XR_A_CUR_ESTD_HOOK | Adult respondents who have ever smoked a hookah, have ever smoked them fairly regularly, and currently smoke every day or some days. | Yes, no | All who have ever used each respective product |
|  |  | R0XR_A_CUR_ESTD_SNUS | Adult respondents who have ever used snus pouches, have ever used them fairly regularly, and currently use every day or some days. | Yes, no | All who have ever used each respective product |
|  |  | R0XR_A_CUR_ESTD_SMKLS | Adult respondents who have ever used smokeless tobacco, have ever used it fairly regularly, and currently use every day or some days. | Yes, no | All who have ever used each respective product |
|  |  |  |  |  |  |

*Denotes derived variables provided by PATH study
