# Supplementary material for: A Population-Level Assessment of Smoking Cessation following a Diagnosis of Tobacco- or Nontobacco-Related Cancer among United States Adults
Source: J Smok Cessat. 2021 Jan 19;2021:6683014. doi: 10.1155/2021/6683014 (PMC8279190; doi:10.1155/2021/6683014)
Supplement: Supplementary 2 — Supplemental Table 1: correlation coefficient matrix for included covariates. Supplemental Table 2: disaggregated comorbid conditions at baseline (Wave I). Supplemental Table 3: disaggregated other tobacco product (OTP) use at baseline (Wave I). [file 6683014.f2.docx]

**Supplemental Tables and Figures**

**Supplemental Table 1** – Correlation coefficient matrix for included covariates

|  | Age | Sex | Charlson | BMI | Marital | Race | Region | Poverty | Education | Attempt2quit | OTP | TTFC |
| --- | --- | --- | --- | --- | --- | --- | --- | --- | --- | --- | --- | --- |
|  |  |  |  |  |  |  |  |  |  |  |  |  |
| Age | 1.00 |  |  |  |  |  |  |  |  |  |  |  |
| Sex | 0.00 | 1.00 |  |  |  |  |  |  |  |  |  |  |
| Charlson | 0.30 | -0.05 | 1.00 |  |  |  |  |  |  |  |  |  |
| BMI | -0.02 | -0.01 | 0.11 | 1.00 |  |  |  |  |  |  |  |  |
| Marital | 0.29 | -0.10 | 0.22 | 0.06 | 1.00 |  |  |  |  |  |  |  |
| Race | -0.05 | 0.05 | -0.04 | 0.04 | -0.08 | 1.00 |  |  |  |  |  |  |
| Region | -0.03 | 0.03 | -0.01 | -0.01 | 0.02 | 0.13 | 1.00 |  |  |  |  |  |
| Poverty | 0.10 | 0.08 | 0.01 | 0.03 | 0.09 | -0.12 | -0.04 | 1.00 |  |  |  |  |
| Education | -0.01 | -0.03 | -0.02 | 0.01 | -0.02 | -0.07 | -0.03 | 0.26 | 1.00 |  |  |  |
| Attempt2quit | -0.02 | -0.05 | 0.05 | 0.01 | 0.00 | 0.06 | 0.01 | -0.01 | 0.01 | 1.00 |  |  |
| OTP | -0.16 | 0.16 | -0.07 | -0.04 | -0.14 | 0.02 | 0.05 | -0.05 | 0.01 | 0.07 | 1.00 |  |
| TTFC | -0.04 | 0.00 | -0.11 | 0.03 | -0.10 | 0.13 | 0.05 | 0.09 | 0.15 | 0.05 | -0.03 | 1.00 |

**Supplemental Table 2** – Disaggregated comorbid conditions at baseline (Wave I)

|  | **n** | **Weighted %** | **95% CI** |
| --- | --- | --- | --- |
| HTN | 1998 | 26.1 | 24.8-27.5% |
| HL | 1431 | 19.5 | 18.5-20.5% |
| CHF | 138 | 1.9 | 1.6-2.2% |
| Stroke | 185 | 2.5 | 2.1-3.0% |
| Heart Dz | 604 | 8.0 | 7.2-8.8% |
| MI | 214 | 3.0 | 2.6-3.6% |
| COPD | 510 | 6.7 | 6.0-7.6% |
| DM2 | 977 | 12.4 | 11.7-13.2% |
| GI Ulcer | 781 | 9.8 | 8.9-10.8% |
| GI Bleed | 297 | 3.7 | 3.2-4.3% |
| Prior history of cancer | 341 | 4.9 | 4.3-5.6% |

**Supplemental Table 3**- Disaggregated other tobacco product (OTP) use at baseline (Wave I)

|  | n | Survey weighted % (95% CI) |
| --- | --- | --- |
| E-products | 714 | 8.3% (7.6-9.1%) |
| Cigars | 308 | 3.7% (3.3-4.2%) |
| Pipe | 110 | 1.2% (1.0-1.5%) |
| Cigarillo | 492 | 5.3% (4.7-5.9%) |
| Filtered Cigars | 291 | 3.6% (3.2-4.0%) |
| Smokeless | 397 | 4.7% (4.1-5.3%) |
| Snus | 96 | 1.1% (0.9-1.4%) |
| Hookah | 309 | 3.0% (2.6-3.5%) |
